# 18.1 MITOCHONDRIAL ALTERATIONS WITHIN THE PYRAMIDAL-PARVALBUMIN CELL MICROCIRCUIT IN THE PREFRONTAL CORTEX IN SCHIZOPHRENIA

**DOI:** 10.1093/schbul/sby014.070

**Published:** 2018-04-01

**Authors:** Jill Glausier

**Affiliations:** University of Pittsburgh

## Abstract

**Background:**

Working memory, a core cognitive function impaired in schizophrenia, depends upon gamma oscillatory neuronal activity in the prefrontal cortex (PFC). Accordingly, individuals with schizophrenia show lower power of gamma oscillations in the PFC during tasks that involve working memory.

Gamma oscillations emerge from the fast and coordinated activity of layer 3 excitatory pyramidal cells and inhibitory parvalbumin (PV) cells. As such, gamma oscillations have a particularly high energetic demand that is met by ATP production via oxidative phosphorylation (OXPHOS) within pyramidal and PV cell mitochondria. PFC layer 3 pyramidal cells have prominent reductions in OXPHOS-related gene pathways in schizophrenia. Importantly, OXPHOS can be regulated by two distinct processes: ATP demand to support neuronal firing, or upstream deficits in OXPHOS enzyme expression.

Layer- and cell type-specific transcriptomic analyses of OXPHOS enzymes and ultrastructural analyses of mitochondrial morphology can help to distinguish between these two possibilities. Reduced ATP demand due to reduced neuronal firing is associated with 1) correlated expression levels of OXPHOS enzyme complexes, 2) lower expression of all subunits comprising Complex IV, the terminal and rate-limiting OXPHOS enzyme complex, and 3) normal mitochondrial morphology. Defective OXPHOS results in 1) elimination of correlated expression of OXPHOS enzyme complexes, 2) variable and inconsistent effects on Complex IV subunit expression, and 3) abnormal mitochondrial morphology.

To determine which upstream factor is likely operative in the illness, we quantified OXPHOS enzyme complex transcripts in layer 3 pyramidal and PV cells, and performed ultrastructural analyses of mitochondrial morphology within pyramidal and PV axon boutons in layer 3 of the PFC in schizophrenia and unaffected comparison subjects.

**Methods:**

For mRNA analyses, frozen tissue sections of area 9 from 36 pairs of schizophrenia and comparison subjects were stained for Nissl substance to identify pyramidal cells, or labeled using immunoperoxidase for aggrecan to identify PV cells. Layer 3 pyramidal and PV somata were dissected using laser capture microdissection. Transcriptome profiling was performed by microarray using Affymetrix GeneChips. Analysis of Complexes I, IV, and V expression in each neuronal population was assessed at q<0.05 in covariate- and multiple comparisons-corrected analyses.

Electron microscopic analyses were performed in area 46 of 2 matched pairs of schizophrenia and comparison subjects. Mitochondria in pyramidal and PV cell boutons were classified as normal or abnormal using established criteria. Chi-square analysis was used to examine whether the percentages of each type differed between groups.

**Results:**

The expression of subunits comprising Complexes I, IV, and V in layer 3 pyramidal and PV cells was significantly lower in subjects with schizophrenia relative to unaffected comparison subjects. In both cell populations, expression of Complexes I, IV, and V were correlated (r=0.8-0.9) in unaffected comparison and schizophrenia subjects. Complex IV subunits showed 11–26% reductions in pyramidal cells, and 7-31% reductions in PV cells in schizophrenia. In both unaffected comparison and schizophrenia subjects, ≥99% of mitochondria in pyramidal cell boutons and ≥97% of mitochondria in PV cell boutons exhibited normal morphology.

**Discussion:**

The current findings are most consistent with the interpretation that lower measures of OXPHOS in schizophrenia reflect lower demand for ATP production due to less neuronal firing.

